# Supplementary material for: Inferring skin–brain–skin connections from infodemiology data using dynamic Bayesian networks
Source: Sci Rep. 2024 May 4;14:10266. doi: 10.1038/s41598-024-60937-3 (PMC11069591; doi:10.1038/s41598-024-60937-3)
Supplement: Supplementary file 1 — Supplementary Legends. [file 41598_2024_60937_MOESM1_ESM.pdf]

Supplementary information

Supplementary Table 1 Mapping between the variables in the Google COVID-19 Public Data Set and the conditions discussed in this paper. When multiple variables map to the same condition, the search query frequencies from those variables were aggregated to give a single overall frequency for the condition.

Supplementary Table 2 Arc strengths for the dynamic Bayesian network model shown in Figure 1. "From" denotes the node at the tail of the arc, "To" denotes the node at the head of the arc, and "Arc strength" is the frequency of the arcs in the bootstrapped models.

Supplementary Figure 1 Proportions of the variance of ACNE, ADHD, ANX, DEP, DER, and SLD explained by their parents in the network shown in Figure 1, unnormalised. This f gure complements Figure 2 in which the proportions are normalised by the total explained variance for the condition.

Supplementary Figure 2 Average relative error (in absolute value) for the missing data imputation algorithms with individual missing values amounting to 2%, 5%, 10% and 20% of the total.

Supplementary Figure 3 Average relative error (in absolute value) for the missing data imputation algorithms with values missing in 1-month batches (4 consecutive weeks) amounting to 2%, 5%, 10% and 20% of the total.

Supplementary Figure 4 Proportion of the variance of each condition explained by the US states, by the counties and by the counties together with the temporal autocorrelation. The average for each of them over all conditions is reported at the bottom.
