# Supplementary figures and images for: Inferring skin–brain–skin connections from infodemiology data using dynamic Bayesian networks
Source: Sci Rep. 2024 May 4;14:10266. doi: 10.1038/s41598-024-60937-3 (PMC11069591; doi:10.1038/s41598-024-60937-3)

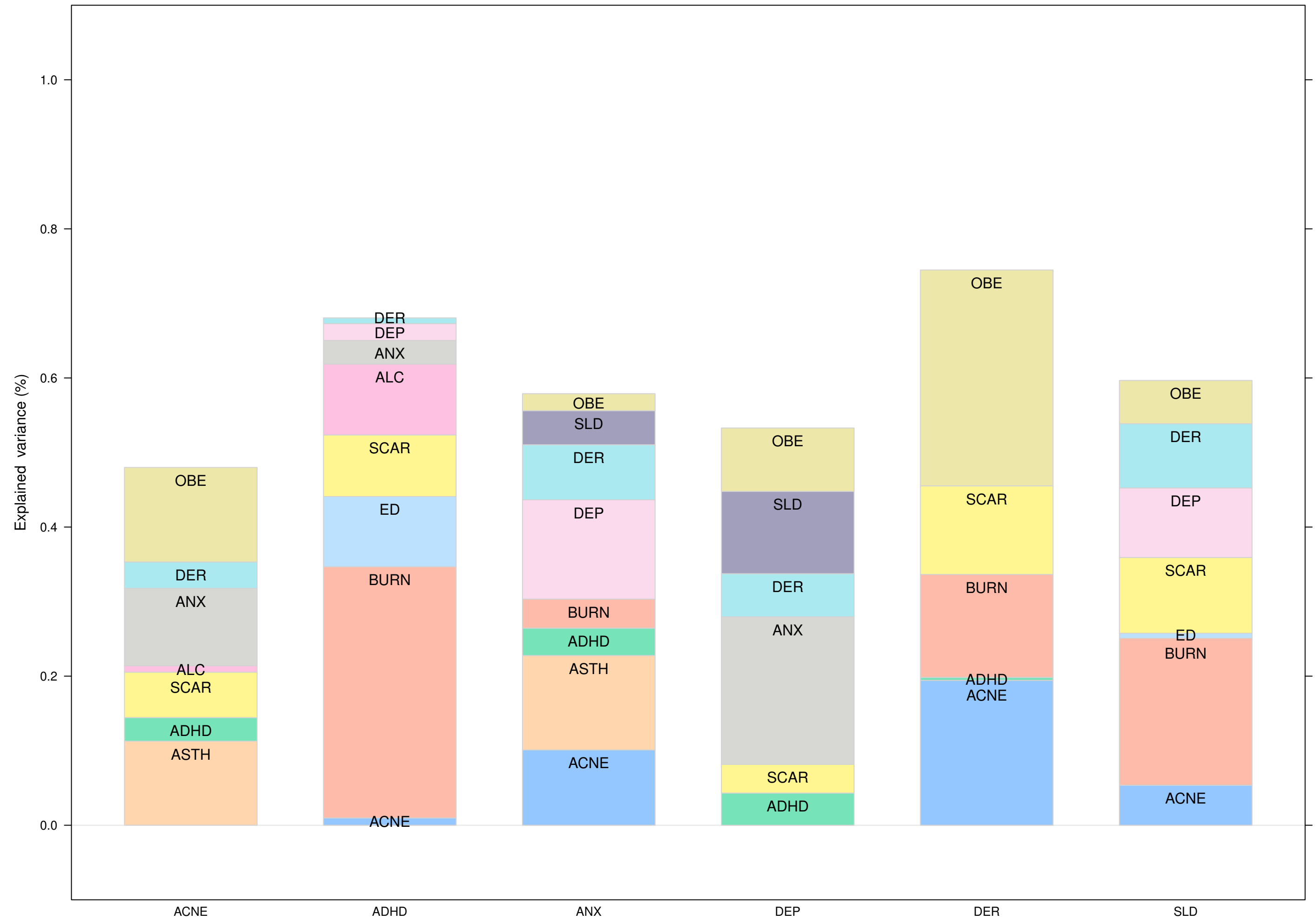

Supplement: Supplementary file 2 — Supplementary Figure 1. [file 41598_2024_60937_MOESM2_ESM.pdf]

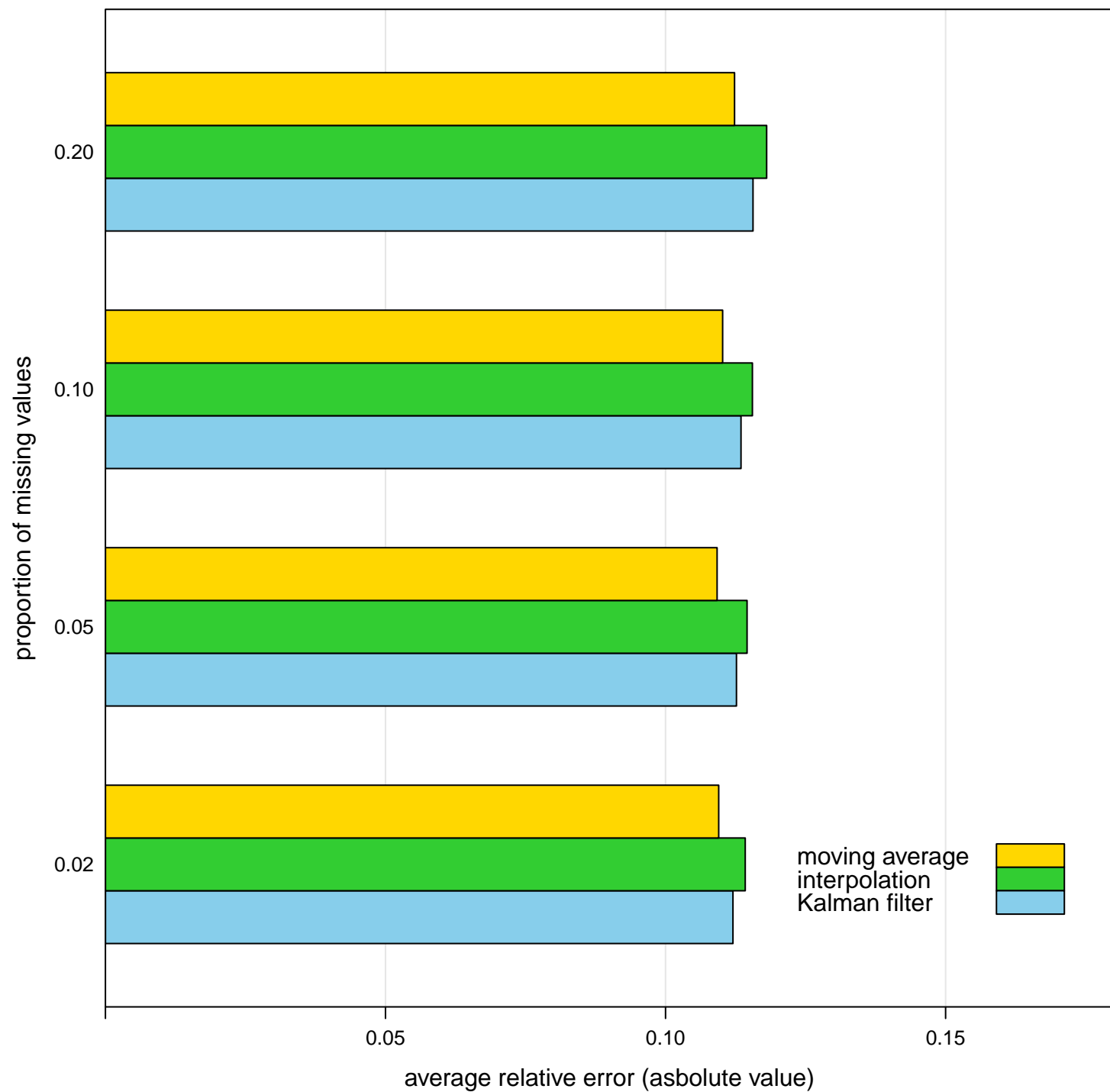

Supplement: Supplementary file 3 — Supplementary Figure 2. [file 41598_2024_60937_MOESM3_ESM.pdf]

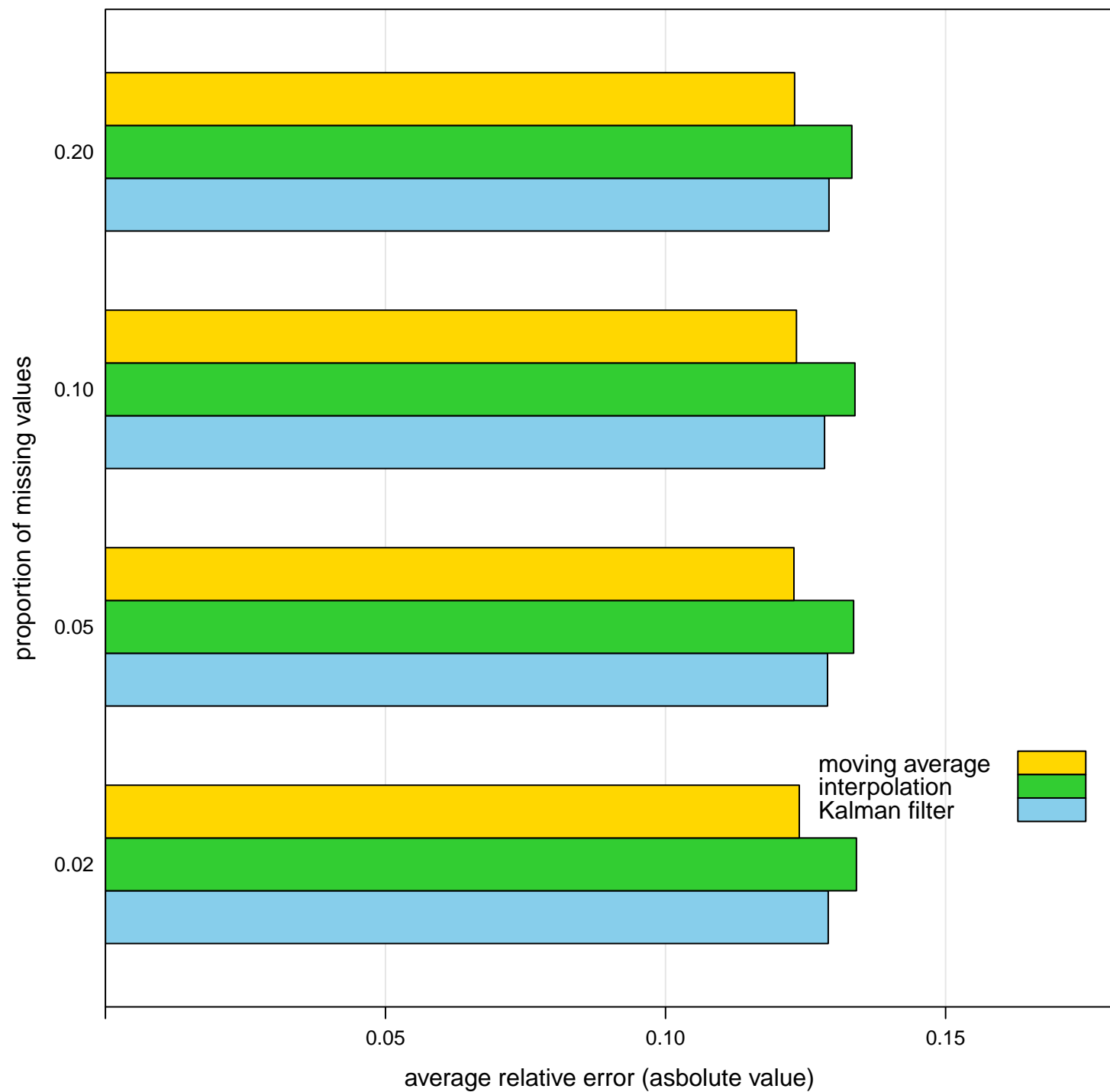

Supplement: Supplementary file 4 — Supplementary Figure 3. [file 41598_2024_60937_MOESM4_ESM.pdf]

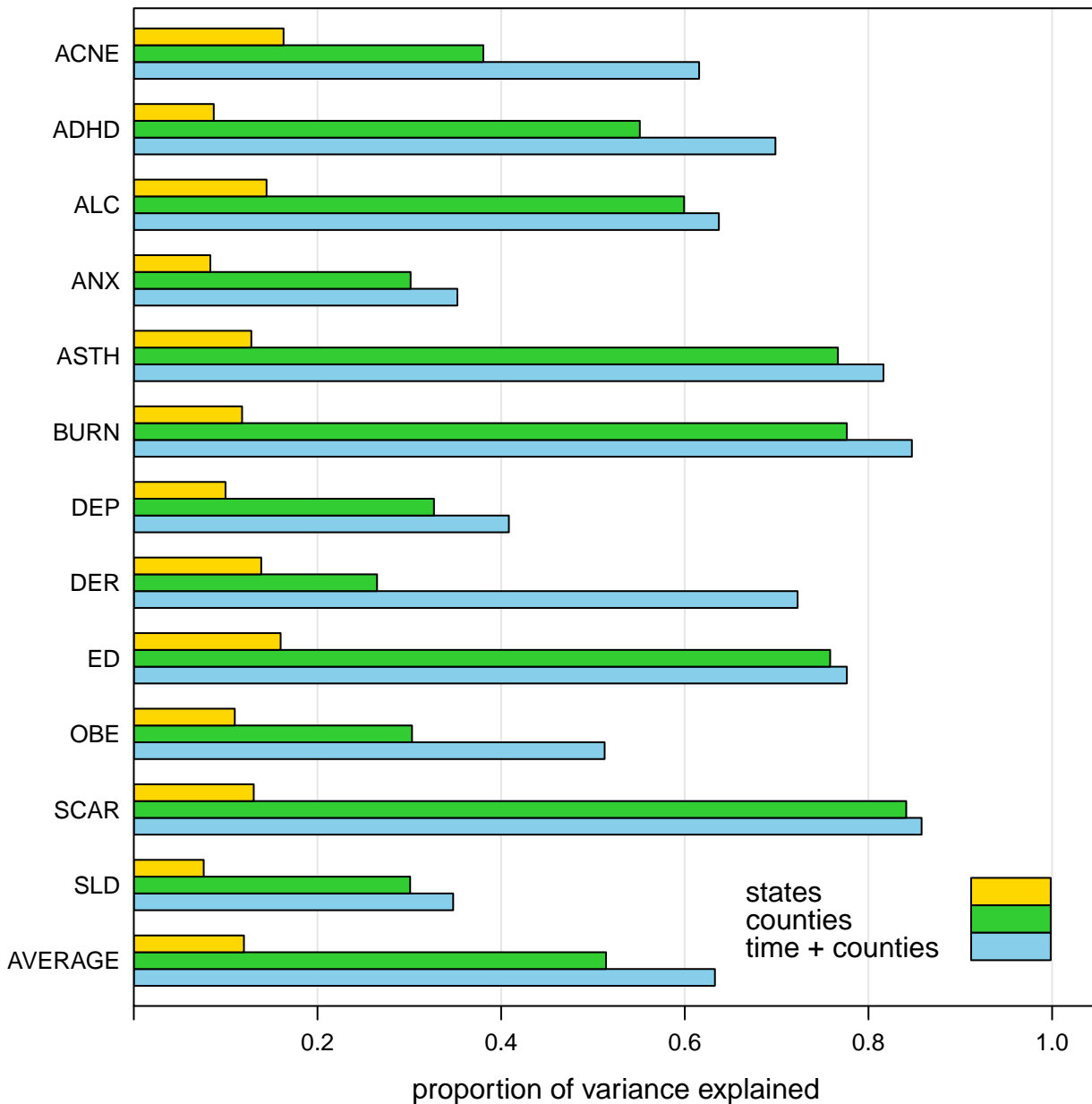

Supplement: Supplementary file 5 — Supplementary Figure 4. [file 41598_2024_60937_MOESM5_ESM.pdf]
